# Supplementary material for: Dynamic anticrack propagation in snow
Source: Nat Commun. 2018 Aug 3;9:3047. doi: 10.1038/s41467-018-05181-w (PMC6076253; doi:10.1038/s41467-018-05181-w)
Supplement: Supplementary file 1 — Supplementary Information [file 41467_2018_5181_MOESM1_ESM.pdf]

# Supplementary Information for: Dynamic anticrack propagation in snow

Gaume et al.

## Supplementary Note 1 - Derivation of return mapping algorithm from plastic flow

The return mapping algorithm is the discrete equivalent to solving for a strain that satisfies the plastic flow rule in Equation 5 of the Methods section and that lies in the CamClay yield surface. In this section first we outline the method of Simo and Meschke [1] to derive the discrete equations from their continuous versions. This procedure starts by assuming there is no plastic flow and a return mapping algorithm is derived from the flow equations that shows how to project back to the yield surface if the assumption of no plastic flow is invalid.

Consider the evolution of $\mathbf{b}^E$ from time $t^n$ to time $t^{n+1} = t^n + \Delta t$. We consider this evolution per particle, and thus it is useful to take a Lagrangian view. We outline the notation used in the Lagrangian view in the Methods section of the paper. Specifically useful here is the flow map $\boldsymbol{\phi} : \Omega^0 \times [0,T] \to \mathbb{R}^d$, and its relation to the deformation gradient $\mathbf{F} = \frac{\partial \boldsymbol{\phi}}{\partial \mathbf{X}}$. Define the time $t^n$ configuration of the material as $\Omega^{t^n} = \left\{ \tilde{\mathbf{x}} = \boldsymbol{\phi}(\mathbf{X}, t^n) \text{ for some } \mathbf{X} \in \Omega^0 \right\}$ and define $\tilde{\boldsymbol{\phi}} : \Omega^{t^n} \times [t^n, T] \to \mathbb{R}^d$ as $\tilde{\boldsymbol{\phi}}(\tilde{\mathbf{x}}, t) = \boldsymbol{\phi}(\boldsymbol{\phi}^{-1}(\tilde{\mathbf{x}}, t^n), t)$. Intuitively, $\tilde{\boldsymbol{\phi}}$ defines the deformation as if the time $t^n$ configuration $\Omega^{t^n}$ of the material is the reference configuration, rather than $\Omega^0$ as in the standard Lagrangian view. This is some times called an updated Lagrangian view. While the deformation gradient $\mathbf{F}$ defines the deformation from the initial configuration ($\Omega^0$) to the time $t$ configuration ($\Omega^t$), the Jacobian $\tilde{\mathbf{F}} = \frac{\partial \tilde{\boldsymbol{\phi}}}{\partial \tilde{\mathbf{x}}}$ defines the deformation from the time $t^n$ configuration ($\Omega^{t^n}$) to the time $t$ configuration ($\Omega^t$), where $t \geq t^n$. Also these are related as $\mathbf{F} = \tilde{\mathbf{F}}\mathbf{F}^n$, or more precisely $\mathbf{F}(\mathbf{X}, t) = \tilde{\mathbf{F}}(\boldsymbol{\phi}(\mathbf{X}, t^n), t)\mathbf{F}(\mathbf{X}, t^n)$ for all $\mathbf{X} \in \Omega^0$.

Define $\mathbf{b}^{E*} = \tilde{\mathbf{F}}^{-1}\mathbf{b}^E\tilde{\mathbf{F}}^{-T}$. Let us consider the difference between the evolution of $\mathbf{b}^{E*}$ and $\mathbf{b}^E$ in absence of plasticity at time $t^n < t < t^{n+1}$. By the definition of $\mathbf{b}^{E*}$, $\frac{D\mathbf{b}^{E*}}{Dt} = -2\dot{\gamma}\tilde{\mathbf{F}}^{-1}\mathbf{G}\tilde{\mathbf{F}}\mathbf{b}^{E*}$, with $\mathbf{G} = \frac{\partial y}{\partial \boldsymbol{\tau}}$, therefore in absence of plasticity $\mathbf{b}^{E*}$ is constant since $\frac{D\mathbf{b}^{E*}}{Dt} = \mathbf{0}$. In contrast, $\mathbf{b}^E\big|_t = \tilde{\mathbf{F}}\Big|_t \mathbf{b}^E\big|_{t^n}\tilde{\mathbf{F}}^T\Big|_t$ in the same case. In other words, $\mathbf{b}^{E*}$ is constant except for the effect of plasticity, but at the same time $\mathbf{b}^E$ would also be stretched by the flow. This isolation of the plastic part allows for a more intuitive discretization. Specifically, if we let $\mathbf{H} = (\dot{\gamma}\tilde{\mathbf{F}}^{-1}\mathbf{G}\tilde{\mathbf{F}})\Big|_{t^{n+1}}$, we have that $\mathbf{b}^{E*}$ approximately satisfies the ODE: $\frac{D\mathbf{Y}}{Dt} = -2\mathbf{H}\mathbf{Y}$ , with $\mathbf{Y}\big|_{t^n} = \mathbf{b}^E\big|_{t^n}$, and we can approximate $\mathbf{b}^{E*}\big|_{t^{n+1}}$ by $\mathbf{Y}\big|_{t^{n+1}} = \exp(-2\Delta t\mathbf{H})\mathbf{b}^E\big|_{t^n} = \exp(-2\Delta\gamma\,\tilde{\mathbf{F}}^{-1}\mathbf{G}\tilde{\mathbf{F}})\Big|_{t^{n+1}}\mathbf{b}^E\big|_{t^n}$ where $\Delta\gamma = \Delta t\,\dot{\gamma} \geq 0$ will be used to enforce the constraint $y(\boldsymbol{\tau}(\mathbf{b}^E\big|_{t^{n+1}})) \leq 0$. Multiplying the approximation by $\tilde{\mathbf{F}}\Big|_{t^{n+1}}$ on the left and $\tilde{\mathbf{F}}^T\Big|_{t^{n+1}}$ on the right, and recalling the definition of $\mathbf{b}^{E*}$, we obtain

$$\begin{aligned}
\mathbf{b}^E\big|_{t^{n+1}} &= \tilde{\mathbf{F}}\Big|_{t^{n+1}}\mathbf{b}^{E*}\big|_{t^{n+1}}\tilde{\mathbf{F}}^T\Big|_{t^{n+1}} \\
&\approx \tilde{\mathbf{F}}\Big|_{t^{n+1}}\exp(-2\Delta\gamma\,\tilde{\mathbf{F}}^{-1}\mathbf{G}\tilde{\mathbf{F}})\Big|_{t^{n+1}}\mathbf{b}^E\big|_{t^n}\tilde{\mathbf{F}}^T\Big|_{t^{n+1}} \\
&= \tilde{\mathbf{F}}\Big|_{t^{n+1}}\tilde{\mathbf{F}}^{-1}\Big|_{t^{n+1}}\exp(-2\Delta\gamma\,\mathbf{G})\big|_{t^{n+1}}\tilde{\mathbf{F}}\Big|_{t^{n+1}}\mathbf{b}^E\big|_{t^n}\tilde{\mathbf{F}}^T\Big|_{t^{n+1}} \\
&= \exp(-2\Delta\gamma\,\mathbf{G})\big|_{t^{n+1}}\tilde{\mathbf{F}}\Big|_{t^{n+1}}\mathbf{b}^E\big|_{t^n}\tilde{\mathbf{F}}^T\Big|_{t^{n+1}}.
\end{aligned}$$

Using the notation $\tilde{\mathbf{b}}^E = \tilde{\mathbf{F}}\Big|_{t^{n+1}}\mathbf{b}^E\big|_{t^n}\tilde{\mathbf{F}}^T\Big|_{t^{n+1}}$, we are looking for a solution pair $\Delta\gamma$ and $\mathbf{b}^E\big|_{t^{n+1}}$ such that

$$\mathbf{b}^E\big|_{t^{n+1}} = \exp\left(-2\Delta\gamma\,\mathbf{G}(\boldsymbol{\tau}(\mathbf{b}^E\big|_{t^{n+1}}))\right)\tilde{\mathbf{b}}^E, \tag{1}$$

and constraint $y(\boldsymbol{\tau}(\mathbf{b}^E\big|_{t^{n+1}})) \leq 0$ is satisfied. Note that $\tilde{\mathbf{b}}^E$ is the elastic strain we would get without the effect of plasticity. For example if $y(\boldsymbol{\tau}(\hat{\mathbf{b}}^E)) \leq 0$, then $\Delta\gamma = 0$ and $\mathbf{b}^E\big|_{t^{n+1}} = \tilde{\mathbf{b}}^E$ is the trivial solution pair and there is

no plastic flow. In this sense, we can see that $\tilde{\mathbf{b}}^E$ can be considered as the trial elastic state obtained without any plastic flow. If this does not satisfy the constraint, $\Delta\gamma$ and $\mathbf{b}^E\big|_{t^{n+1}}$ must be defined to "project" $\tilde{\mathbf{b}}^E$ to $\mathbf{b}^E\big|_{t^{n+1}}$.

We use this process to define the projection. $\tilde{\mathbf{F}}\mathbf{F}^E$ is considered the trial elastic state, one obtained in the absence of plastic flow. Thus, $\tilde{\mathbf{b}}^E = \tilde{\mathbf{F}}\mathbf{b}^E\tilde{\mathbf{F}}^T$ and we seek the solution of Equation 1 to define the projection to $\mathbf{b}^E\big|_{t^{n+1}}$. This can be done most easily by considering the singular value decomposition of $\tilde{\mathbf{F}}\mathbf{F}^E$.

If the singular value decomposition of $\tilde{\mathbf{F}}\mathbf{F}^E$ is given by $\tilde{\mathbf{F}}\mathbf{F}^E = \mathbf{U}\tilde{\boldsymbol{\Sigma}}\mathbf{V}^T$, then $\tilde{\mathbf{b}}^E = \mathbf{U}\tilde{\boldsymbol{\Sigma}}^2\mathbf{U}^T$. It can be shown that $\mathbf{U}$ diagonalizes $\mathbf{G}(\boldsymbol{\tau}(\mathbf{b}^E\big|_{t^{n+1}}))$ and $\mathbf{b}^E\big|_{t^{n+1}}$ (i.e. $\mathbf{G}(\boldsymbol{\tau}(\mathbf{b}^E\big|_{t^{n+1}})) = \mathbf{U}\hat{\mathbf{G}}(\boldsymbol{\Sigma})\mathbf{U}^T$, and $\mathbf{b}^E\big|_{t^{n+1}} = \mathbf{U}\boldsymbol{\Sigma}^2\mathbf{U}^T$ with $\hat{\mathbf{G}} = \frac{\partial y}{\partial \hat{\boldsymbol{\tau}}}$ and $\hat{\boldsymbol{\tau}} = \mathbf{U}^T\boldsymbol{\tau}(\mathbf{b}^E\big|_{t^{n+1}})\mathbf{U}$), then we may write (1) as

$$\begin{aligned}\mathbf{U}\boldsymbol{\Sigma}^2\mathbf{U}^T &= \exp\left(-2\Delta\gamma\mathbf{U}\hat{\mathbf{G}}(\boldsymbol{\Sigma})\mathbf{U}^T\right)\mathbf{U}\tilde{\boldsymbol{\Sigma}}^2\mathbf{U}^T \\ &= \mathbf{U}\exp\left(-2\Delta\gamma\hat{\mathbf{G}}(\boldsymbol{\Sigma})\right)\tilde{\boldsymbol{\Sigma}}^2\mathbf{U}^T.\end{aligned}$$

Multiplying both sides of the previous equation by $\mathbf{U}^T$ on the left and by $\mathbf{U}$ on the right, and taking log results in

$$2\log(\boldsymbol{\Sigma}) = -2\Delta\gamma\hat{\mathbf{G}}(\boldsymbol{\Sigma}) + 2\log\left(\tilde{\boldsymbol{\Sigma}}\right). \tag{2}$$

The model that we choose uses the Hencky-strain as a measure of deformation. By defining

$$\hat{\boldsymbol{\epsilon}}^{\text{tr}} := \log\tilde{\boldsymbol{\Sigma}} \quad \text{and} \quad \hat{\boldsymbol{\epsilon}}^{n+1} := \log\boldsymbol{\Sigma}, \tag{3}$$

we may simplify and rearrange Equation (2)

$$\hat{\boldsymbol{\epsilon}}^{\text{tr}} - \hat{\boldsymbol{\epsilon}}^{n+1} = \Delta\gamma\hat{\mathbf{G}}. \tag{4}$$

This is our discrete flow rule. In the return mapping algorithm, we want to solve for $\hat{\boldsymbol{\epsilon}}^{n+1}$ satisfies Equation (4) subject to the constraint

$$y(\hat{\boldsymbol{\tau}}(\hat{\boldsymbol{\epsilon}}^{n+1})) \leq 0. \tag{5}$$

Solving Equation (4) and (5) can be seen as a ray-ellipse intersection problem due to the ellipsoid shape of our CamClay yield surface.

## Supplementary Note 2 - Shear behavior of the weak layer

We describe and discuss the mechanical behavior of the weak layer in shear by simulating an unconfined shear-test (simulation setup corresponds to experiment n° 2 with a wall horizontal velocity of 0.01 m.s$^{-1}$, Supplementary Figure 1). After reaching the shear strength of the weak layer, significant softening is observed followed by weak layer collapse (almost 20% of weak layer thickness). Then, the weak layer has a pure frictional behavior with a residual shear strength. This mechanical behavior is very similar to what has been reported in laboratory experiments of shear failure of snow [2] and what has been assumed in interfacial shear models [3, 4]. In particular, the so-called critical sliding displacement (displacement $\delta_s$ associated to the softening zone) has the same order of magnitude as in [3] and [2, 4] ($\delta_s = 3.5$ mm in [3], $\delta_s = 2$ mm in [2, 4] and $\delta_s \sim 2$ mm in our simulation).

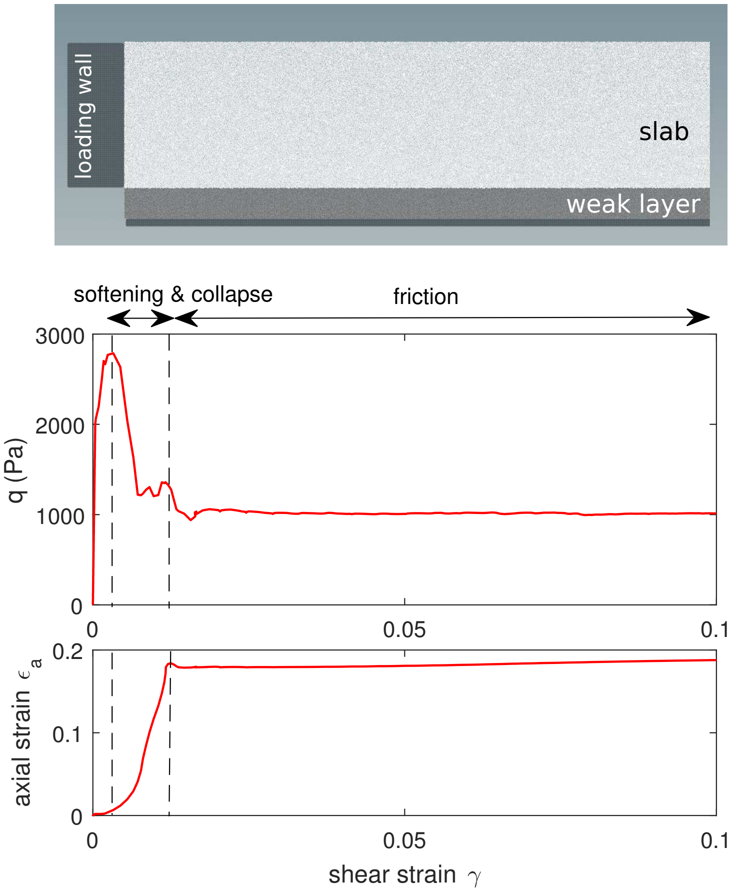


Supplementary Figure 1: Top: Shear test simulation of the weak layer under slab normal load. The setup corresponds the PST simulation of experiment n° 2. The wall on the left is displaced along the $x$-direction with a constant speed of 0.01 m.s$^{-1}$. Middle: Mises equivalent stress $q$ vs shear strain $\gamma$ in a pure shear test (displacement controlled). Bottom: Axial strain $\epsilon_a$ vs shear strain $\gamma$.

# Supplementary Note 3 - Influence of mesh resolution

We show the effect of mesh refinement on the amount of volumetric collapse of the weak layer. The collapse is controlled by the hardening rule (point (2*) to (3*) in Figure 1 of the paper). It characterizes the thickness of the anticrack (or compacting shear band) and can be evaluated by computing the change in volumetric plastic deformation as $1 - J_P$ with $J_P = \det(\mathbf{F}^P)$ $(0 < J_P \leq 1)$. The collapse height $h_c$ can be evaluated by computing $h_c = (1 - J_P)D_{\mathrm{wl}}$. Here, we simulated the shear test presented in Supplementary Note 3 for different mesh resolutions and for different values of the parameters of our hardening rule. Supplementary Figure 2 shows that our model is consistent with mesh refinement as $1 - J_P$ is almost not influenced by the mesh resolution.

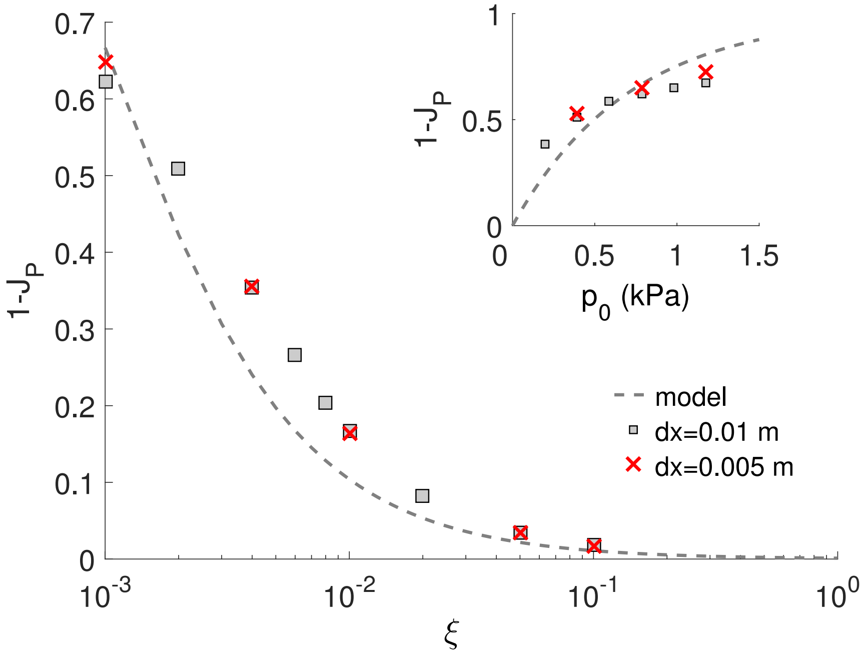


Supplementary Figure 2: Influence of mesh resolution $dx$, hardening factor $\xi$ and consolidation pressure $p_0$ (induced by the slab load) on $1 - J_P$. $p_0$ was modified by varying slab density. The dashed line (model) is computed directly from the theoretical hardening rule (Equation 4 of the paper).

# Supplementary References


[1] J. Simo and G. Meschke. A new class of algorithms for classical plasticity extended to finite strains. application to geomaterials. *Comput. Mech.*, 11(4):253–278, 1993.

[2] D.M. McClung. Direct simple shear tests on snow and their relation to slab avalanche formation. *J. Glaciol.*, 19(81):101–109, 1977.

[3] Z.P. Bazant, Zi G, and D.M. McClung. Size effect law and fracture mechanics of the triggering of dry snow slab avalanches. *J. Geophys. Res.*, 2003.

[4] J. Gaume, G. Chambon, N. Eckert, and M. Naaim. Influence of weak-layer heterogeneity on snow slab avalanche release: Application to the evaluation of avalanche release depths. *J. Glaciol.*, 59(215):423–437, 2013.
