# Supplementary material for: Dynamic anticrack propagation in snow
Source: Nat Commun. 2018 Aug 3;9:3047. doi: 10.1038/s41467-018-05181-w (PMC6076253; doi:10.1038/s41467-018-05181-w)
Supplement: Supplementary file 3 — Description of Additional Supplementary Information [file 41467_2018_5181_MOESM3_ESM.pdf]

Description of additional supplementary files for: Dynamic anticrack propagation in snow

Gaume et al.

Description of Supplementary Movies

We describe here the supplementary movies, the computational time as well as system dimensions and boundary conditions. Note that the computational time is not only a function of the number of particles but also depends on other parameters such as the elastic modulus and the loading speed (e.g. saw speed). Simulations were performed on a workstation which has 24 i7 Intel CPU cores and 128 Gb of RAM. In all simulations, the initial position of MPM particles were sampled using Poisson Disk distribution.

Supplementary Movie 1 - Comparison between model and experiment n° 1:

Experimental (top) and numerical (bottom) results for Exp 01. The displacement field is shown on the left while the evolution of the average vertical displacement is shown on the right. Concerning boundary conditions, the bottom of the weak layer is fixed. This simulation requires 25 minutes ($\sim$ 46000 particles, 120 fps).

Supplementary Movie 2 - Comparison between model and experiment n° 2:

Experimental (top) and numerical (bottom) results for Exp 02. The displacement field is shown on the left while the evolution of the average vertical displacement is shown on the right. Concerning boundary conditions, the bottom of the weak layer is fixed. This simulation needs 35 minutes ($\sim$ 79000 particles, 120 fps).

Supplementary Movie 3 - Comparison between model and experiment n° 3:

Experimental (top) and numerical (bottom) results for Exp 03. The displacement field is shown on the left while the evolution of the average vertical displacement is shown on the right. Concerning boundary conditions, the bottom of the weak layer is fixed. This simulation requires 15 minutes ($\sim$ 41000 particles, 120 fps).

Supplementary Movie 4 - 2D simulation of the remote-triggering of a slab avalanche:

Result of a 2D MPM simulation of remote triggering of a slab avalanche by a skier (simulated as a snowman) including the subsequent flow of the avalanche and burial of the skier. The red color represents failed particles of the weak layer and the dark blue color represents failed particles in the slab. Additional Figure 1 shows the system dimensions and boundary conditions. The slab depth D is constant. This simulation requires 4 hours ($\sim$ 2 million particles, 48 fps).

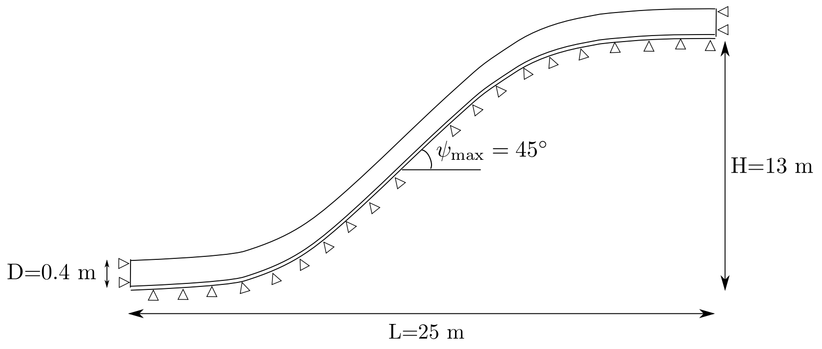

Additional Figure 1: System dimensions for the 2D slope simulation. Triangles represent fixed boundary conditions.

Supplementary Movie 5 - Zoom on the crown fracture:

Supplementary Movie 4 with a zoom and slowmotion (1200 fps) on the crown fracture highlighting the branching of slab fracture from the bottom of the slab at the interface with the weak layer. The red color represents failed particles of the weak layer and the dark blue color represents failed particles in the slab.

Supplementary Movie 6 - 3D simulation of the remote-triggering of slab avalanche:

Result of a 3D MPM simulation of remote triggering of a slab avalanche by a skier (simulated as a snowman) including the subsequent flow of the avalanche and burial of the skier. Additional Figure 2 shows the system dimensions and boundary conditions. The geometry was chosen to mimic a concave slope with a maximum snow depth in the middle of the path. It was reported by [1] that this type of slope shape was most commonly associated with avalanches. Slab depth D is constant with respect to slope angle. This simulation requires 2 days ($\sim$ 10 million particles, 48 fps). The particles where rendered using the software Houdini. In this simulations, the flanks meet the crown at a 90 degrees angle because the failure reached the boundary condition of our system on the sides. This is due to the system dimensions and the homogeneity of the system properties.

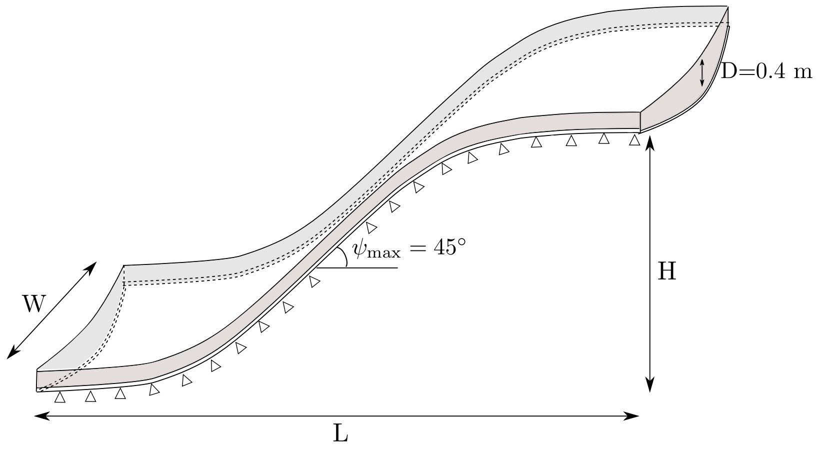

Additional Figure 2: System dimensions for the 3D slope simulations (Supplementary Movies 6 and 7). For Supplementary Movie 6, $L = 18$ m, $H = 7$ m and $W = 10$ m. For Supplementary Movie 7, $L = 25$ m, $H = 10$ m and $W = 15$ m. The bottom of the weak layer and the greyed zones (left, right, top and bottom side walls) are fixed boundary conditions.

Supplementary Movie 7 - Large 3D simulation of the remote-triggering of slab avalanche with spatial variability of snow depth:

Same as in Supplementary Movie 6 but with a larger system size and with spatial variability of snow depth (+/- $0.25D$ with a correlation length of 5 m). This simulation requires 5 days ($\sim$ 30 million particles, 48 fps). The particles where rendered using the software Houdini.

Additional References

[1] I. Vontobel, S. Harvey, and R.S. Purves. Terrain analysis of skier-triggered avalanche starting zones. *Proceedings of the International Snow Science Workshop ISSW, Grenoble, France, 7 - 11 October 2013*, 2013.
